# Impact of enzyme replacement therapy on survival in adults with Pompe disease: results from a prospective international observational study

**DOI:** 10.1186/1750-1172-8-49

**Published:** 2013-03-27

**Authors:** Deniz Güngör, Michelle E Kruijshaar, Iris Plug, Ralph B D’Agostino, Marloes LC Hagemans, Pieter A van Doorn, Arnold JJ Reuser, Ans T van der Ploeg

**Affiliations:** 1 Center for Lysosomal and Metabolic Diseases, Erasmus MC University Medical Center, Rotterdam, the Netherlands; 2 Department of Paediatrics, Erasmus MC University Medical Center, Rotterdam, the Netherlands; 3 Department of Mathematics and Statistics, Boston University, Harvard Clinical Research Institute, Boston, MA, USA; 4 Department of Neurology, Erasmus MC University Medical Center, Rotterdam, the Netherlands; 5 Department of Clinical Genetics, Erasmus MC University Medical Center, Rotterdam, the Netherlands; 6 Department of Paediatrics, Center for Lysosomal and Metabolic Diseases, Erasmus MC University Medical Center – Sophia Children’s Hospital, P.O. Box 2060, Rotterdam, CB 3000, the Netherlands

**Keywords:** Pompe disease, Survival, Acid maltase deficiency, Lysosomal storage disease, Glycogen storage disease type II, Enzyme replacement therapy, Alglucosidase alfa

## Abstract

**Background:**

Pompe disease is a rare metabolic myopathy for which disease-specific enzyme replacement therapy (ERT) has been available since 2006. ERT has shown efficacy concerning muscle strength and pulmonary function in adult patients. However, no data on the effect of ERT on the survival of adult patients are currently available. The aim of this study was to assess the effect of ERT on survival in adult patients with Pompe disease.

**Methods:**

Data were collected as part of an international observational study conducted between 2002 and 2011, in which patients were followed on an annual basis. Time-dependent Cox’s proportional hazards models were used for univariable and multivariable analyses.

**Results:**

Overall, 283 adult patients with a median age of 48 years (range, 19 to 81 years) were included in the study. Seventy-two percent of patients started ERT at some time during follow-up, and 28% never received ERT. During follow-up (median, 6 years; range, 0.04 to 9 years), 46 patients died, 28 (61%) of whom had never received ERT. After adjustment for age, sex, country of residence, and disease severity (based on wheelchair and ventilator use), ERT was positively associated with survival (hazard ratio, 0.41; 95% CI, 0.19 to 0.87).

**Conclusion:**

This prospective study was the first to demonstrate the positive effect of ERT on survival in adults with Pompe disease. Given the relatively recent registration of ERT for Pompe disease, these findings further support its beneficial impact in adult patients.

## Background

Pompe disease (glycogen storage disease type II, acid maltase deficiency) is a rare metabolic myopathy caused by a deficiency of lysosomal acid α-glucosidase (GAA), resulting in the intralysosomal accumulation of glycogen. Pompe disease is characterized by a progressive loss of muscle strength and respiratory function and is inherited in an autosomal recessive manner [1]. Historically, supportive care has been the mainstay of treatment. Following decades of research, enzyme replacement therapy (ERT) for Pompe disease was approved in Europe and the United States in 2006, prompting a new era in the treatment of this disease. This was the first disease-specific treatment for an inherited muscular disorder, [2] consisting of the intravenous administration of recombinant human GAA (alglucosidase alfa) [3].

The therapeutic efficacy of alglucosidase alfa was first demonstrated in classic infantile Pompe disease [3,4]. Patients with classic infantile Pompe disease present with symptoms shortly after birth and develop significant, progressive hypertrophic cardiomyopathy and loss of skeletal muscle function within months if they remain untreated [5]. Cardiorespiratory failure is the primary cause of mortality, typically occurring within the first year of life [5]. With the introduction of ERT, survival in patients with classic infantile Pompe disease has increased significantly; [5] the oldest infants treated with ERT are now 14 years of age [4,6].

The rapid decline in classic infantile Pompe disease is explained by the complete lack of GAA activity [1]. However, the majority of patients with Pompe disease express some residual GAA activity, leading to a spectrum of disease presentations [7]. In adults, the disease progresses more slowly, with loss of ambulation and wheelchair and respirator dependency developing in later stages of the disease at varying ages [8]. Their primary cause of death is respiratory failure [1,7].

Trials of ERT in adults were initiated much later than those in infants. The first and only placebo-controlled randomized trial started at the end of 2005 and included 90 patients. Over a period of 78 weeks, treatment with alglucosidase alfa resulted in an improved walking distance on the six-minute walk test and stabilization of pulmonary function, meeting both primary endpoints of the trial [9]. Since 2006, more adult patients are gradually being treated [10-15], but no studies to date have assessed the impact of ERT on survival in adults.

As an ultra-orphan drug, alglucosidase alfa is an expensive treatment; in several countries around the world, its high cost and lifelong administration have led to a debate on its reimbursement in adults. Survival is a key parameter in this discussion. Our centre has systematically collected data on patients with Pompe disease since 2002, before the approval of ERT. This activity has provided a unique set of long-term survey-based data allowing the evaluation of a large international patient population, both treated and untreated. To date, this database has the longest consistent follow-up providing information on patients with Pompe disease prior to and following ERT initiation. Findings from this survey recently revealed that untreated adults with Pompe disease have higher mortality than the general population [16]. Age, wheelchair and ventilator dependency, and level of handicap appeared to be the main indicators of lower life expectancy [16].

The aim of the current study was to use data collected from the same patient survey to explore the potential effect of ERT on survival in adults with Pompe disease. We report here the results of this prospective, international observational study.

## Methods

### The International Pompe Association/Erasmus MC Pompe Survey

The International Pompe Association (IPA)/Erasmus MC Pompe Survey, an ongoing international observational follow-up study on the clinical course of patients with Pompe disease, has continually enrolled patients since May 2002. The design of this prospective study was described elsewhere [17,18]. Patients were recruited through patient organizations affiliated with the IPA from Australia, Canada, Germany, the Netherlands, the United Kingdom, the United States, and a small number of patients from other countries. Dutch patients included in the analyses participated either directly through Erasmus MC (the designated centre for all known patients with Pompe disease in the Netherlands) or through the Dutch patient organization. Enrolment was independent of the stage of disease and the age of disease onset. The IPA/Erasmus MC Pompe Survey covers the entire spectrum of the disease and is representative of the adult Pompe population [19].

Information was collected through annual questionnaires, which asked patients about their medical history, current disease status, use of care, and quality of life. For Dutch patients, additional data were obtained during regular clinical evaluations at Erasmus MC, producing more frequent follow-up measurements than for other patients. The date of the last completed questionnaire before September 2011 was considered as the date of last follow-up, or - for the Dutch group - the date of the last visit if this came last. When questionnaires were not returned it was investigated whether the patient had died. The date of death was either reported, or estimated to be halfway between the date of completion of the last questionnaire and the date at which the next questionnaire should have been completed.

All research protocols were approved by the Medical Ethics Committee of Erasmus MC and/or the Central Committee on Research Involving Human Subjects. Written informed consent was obtained from all patients.

The current study included only patients 18 years of age or older at study entry and used data collected until September 2011. At that time, the database included information on 369 participants 18 years of age or older at enrolment. Patients were excluded from the analysis if only baseline data were available (n=71), if they had started receiving ERT before study enrolment (n=13), or if relevant baseline data were missing (n=2).

### Statistical analysis

Data describing the patients’ characteristics are presented as medians and ranges. Patient characteristics were compared using the Mann–Whitney test or the χ^2^ test. Survival time was assessed from the date of study entry until the date of last follow-up or until death. The association between overall survival of adult patients and treatment with ERT was estimated using time-dependent Cox proportional hazard regression models, both for univariable as well as multivariable analyses. The following covariates were considered and chosen a priori: age, sex, disease severity (based on wheelchair and ventilator use), and country of residence. The results are presented as hazard ratios (HRs) with 95% confidence intervals (CIs).

Two models were generated to describe the relationship between ERT and overall survival in adult patients with Pompe disease. In both models, ERT was included as a time-dependent covariate that took the value 0 before the start of treatment and switched to 1 at the start of treatment. As long as patients were not receiving ERT, they contributed to the untreated group and acted as controls for the treated patients during the treatment period. In the primary model (model 1a), in addition to ERT, age categories and disease severity were also modelled as time-dependent covariates and hence were updated at the start of ERT. An intent-to-treat approach was adopted in which patients who discontinued treatment were considered to have remained in the treatment group until the end of follow-up. The sensitivity of these analyses to including age and severity as time-dependent covariates was investigated using a second model with ERT as a time-dependent variable only (model 2a). Further to the intent-to-treat approach, the primary and secondary analyses were also conducted to account for the actual treatment duration. In these models, all patients who discontinued treatment were censored at the time of discontinuation (models 1b and 2b). The validity of the proportional hazards assumption was assessed by examining plots of the cumulative hazard function on a linear and logarithmic scale, stratified by categories of the covariates. Proportionality was assumed if the curves were parallel.

Statistical tests were conducted using SPSS for Windows (version 17; SPSS Inc., Chicago, IL) and SAS (version 9·2; SAS Institute Inc., Cary, NC). A P-value ≤0.05 was considered statistically significant.

## Results

### Patient characteristics

Overall, 283 adult patients with Pompe disease (77% of those enrolled) were eligible for analysis. The baseline characteristics of these patients are shown in Table 1. Seventy-two percent of patients started ERT at some time during follow-up, and 28% never received ERT. The median age of the patients at study entry was 48 years (range, 19 to 81 years), with a median disease duration of 9 years (range, 0 to 32 years). Fifty-three percent of the patients were women.

**Table 1:** Patient characteristics and follow-up*

**Characteristics**	**n = 283**
Female, n (%)	149 (53)
Median age at study entry, years (range)	48 (19–81)
Median age at diagnosis, years (range)	38 (1–72)
Median disease duration at study entry, years (range)	9 (0–32)
Country of residence, n (%)	
Netherlands	109 (39)
United Kingdom	23 (8)
United States	71 (25)
Germany	48 (17)
Other†	32 (11)
Disease severity at study entry, n (%)	
No wheelchair use or respiratory support^‡^	134 (47)
Wheelchair use	37 (13)
Use of respiratory support	42 (15)
Both wheelchair use and respiratory support	70 (25)
Median follow-up time, years (range)	6 (0.04-9)
ERT during the course of the study, n (%)	204 (72)
Median ERT duration, years (range)	4 (0.2-8)
Median age at start of ERT, years (range)	51 (24–76)
Died during follow-up, n (%)	46 (16)
Median age at death, years (range)	59 (23–86)

* Continuous variables are expressed as median (range). Categorical variables are expressed as n (%). ERT denotes enzyme replacement therapy.

† Including patients from Australia and Canada.

‡ Respiratory support includes partial and full-time invasive and non-invasive respiratory support.

Table 2 shows the characteristics of the ERT and the non-ERT groups at the start of ERT and at study entry. For patients who received ERT during follow-up, the median age at the start of ERT was comparable to the median age at study entry of patients who never received ERT. Differences in sex, age at diagnosis, disease duration, and disease severity (based on use of wheelchair and respiratory support) between treated patients at the start of ERT and untreated patients at enrolment were not significant, whereas country of residence differed with borderline statistical significance.

**Table 2:** Patient characteristics for the ERT group at study entry or start of ERT and for untreated patients at study entry*

	**ERT group (n = 204)**		**Non-ERT group (n = 79)**	**P Value†**
**Characteristics**	**At study entry**	**At start of ERT**	**At study entry**	
Female, n (%)	104 (51)	104 (51)	45 (57)	0.37
Median age at study entry/start of ERT, years (range)	47 (19–73)	51 (24–76)	51 (20–81)	0.48
Median age at diagnosis, years (range)	38 (1–72)	38 (1–72)	42 (2–67)	0.45
Median disease duration at study entry/start of ERT, years (range)	7 (0–31)	11 (0.2-33)	12 (0–32)	0.75
Country of residence, n (%)				0.05
Netherlands	86 (42)	86 (42)	23 (29)	
United Kingdom	18 (9)	18 (9)	5 (6)	
United States	44 (22)	44 (22)	27 (34)	
Germany	37 (18)	37 (18)	11 (14)	
Other‡	19 (9)	19 (9)	13 (17)	
Disease severity at study entry/start of ERT, n (%)				0.45
No wheelchair use or respiratory support§	99 (49)	70 (34)	35 (44)	
Wheelchair use	26 (13)	37(18)	11 (14)	
Use of respiratory support	31(15)	29 (14)	11 (14)	
Both wheelchair use and respiratory support	48 (24)	68 (33)	22 (28)	
Median follow-up time from study entry/start of ERT, years (range)	7 (1–9)	4 (0.2-8)	4 (0.04-9)	0.05
Died during follow-up, n (%)	18 (9)	18 (9)	28 (35)	<0.001

* Continuous variables are expressed as median (range). Categorical variables are expressed as n (%). ERT denotes enzyme replacement therapy.

† P value for differences between ever-treated patients at the start of ERT and never-treated patients (at study entry) as assessed with a Mann–Whitney test or the *χ*^2^ test.

‡ Including patients from Australia and Canada.

§ Respiratory support includes partial and full-time invasive and non-invasive respiratory support.

During the 1676 person-years of follow-up (median, 6 years; range, 0.04 to 9 years), for 46 patients a death confirmation was received from the patient organization, the family or the treating physician. Twenty-eight (61%) of these patients were in the non-ERT group. The median age at death was 59 years (range, 23 to 86 years). Compared with the total patient population, the deceased patients were more severely affected by Pompe disease at study entry. Thirty-seven of the 46 deceased patients (80%) used either a wheelchair or a ventilator or both at study entry compared with 53% of the overall patient population. Causes of death in 21 of the 46 cases were (n=16) or could be (n=5) related to Pompe disease (e.g., respiratory insufficiency). In the remaining 25 cases, the cause of death was either unknown (n=20) or not related to Pompe disease (n=5). Of the 28 patients who died without ever having received ERT, 19 died before or in the year that ERT obtained approval (2006), and 9 died later.

Nineteen of the 204 patients who received ERT stopped treatment during follow-up. The median treatment duration in these patients was 1.4 years (range, 0.2 to 4.7 years), and the median time after stopping treatment until the end of follow-up was 1.2 years (range, 0.05 to 4.0 years). Reasons for discontinuation were related to allergic type reactions/adverse events (n = 10), lack of treatment effect (n = 4), pregnancy (n = 2), and unknown (n = 3). Four of the patients who stopped treatment died, including 3 who had received ERT for less than 1.5 years. Of these 4 patients, 1 died 6 weeks after stopping treatment, and the other 3 patients died between 1 and 2.5 years after stopping treatment.

### Association between Ert and survival

**Figure 1:**
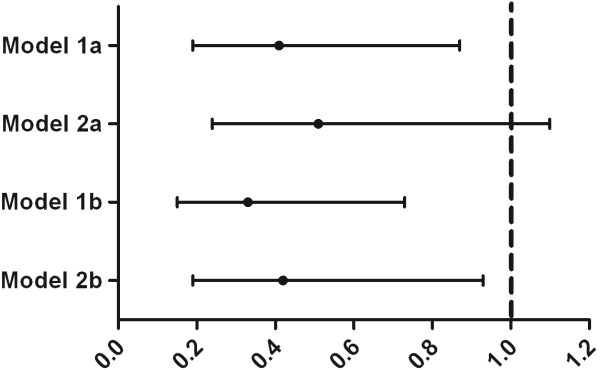
**Adjusted Hazard Ratios of the Different Models Describing the Relationship Between ERT and Survival.** Model 1a: Intent-to-treat approach with enzyme replacement therapy (ERT), age categories, and disease severity as time-dependent covariates. Model 2a: Intent-to-treat approach with only ERT as time-dependent covariate. Model 1b: Analysis excluding person-time after discontinuation of treatment with ERT, age categories, and disease severity as time-dependent covariates. Model 2b: Analysis excluding person-time after discontinuation of treatment with only ERT as time-dependent covariate.

Table 3 summarizes the results from the primary multivariable Cox proportional hazard regression model (model 1a). ERT was shown to be positively associated with survival (HR, 0.46; 95% CI, 0.22 to 0.95) in the univariable analysis. After adjustment for age, sex, country of residence, and disease severity, the HR for ERT was 0.41 (95% CI, 0.19 to 0.87). The model using only ERT as a time-dependent covariate (model 2a) produced an HR of 0.51 (95% CI, 0.24 to 1.10). The analyses in which the patients who discontinued treatment were included until discontinuation resulted in HRs of 0.33 (95% CI, 0.15 to 0.73) and 0.42 (95% CI, 0.19 to 0.93) for models 1b and 2b, respectively. The Forest plot in Figure 1 illustrates the HRs and 95% CIs of all models.

**Table 3:** Risk of death for 283 adults with Pompe disease applying time-dependent cox regression*

	**Model 1a†**		
**Time-dependent cox regression model**	**HR**	**95% CI**	**P Value**
ERT‡	0.41	0.19 to 0.87	0.02
Age (in quartiles)			0.14
<37 years (ref)	1		
37-48 years	1.26	0.38 to 4.12	0.71
48-57 years	1.42	0.44 to 4.61	0.56
≥57 years	2.57	0.86 to 7.72	0.09
Sex	1.01	0.55 to 1.87	0.98
Disease severity			0.001
No wheelchair use or respiratory support ‡	1		
Wheelchair use	2.87	0.98 to 8.36	0.05
Use of respiratory support	2.05	0.62 to 6.77	0.24
Both wheelchair use and respiratory support	5.32	2.25 to 12.56	<0.001
Country of residence			0.13
Netherlands (ref)	1		
United Kingdom	1.35	0.46 to 3.95	0.58
United States	2.14	1.01 to 4.55	0.05
Germany	0.62	0.19 to 1.95	0.41
Other§	1.23	0.42 to 3.63	0.70

* ERT denotes enzyme replacement therapy and HR hazard ratio.

† Intent-to-treat approach with ERT, age categories, and disease severity as time-dependent covariates.

‡ Values for ERT were derived after adjustment for age, sex, disease severity, and country of residence.

§ Including patients from Australia and Canada.

‡Respiratory support includes partial and full-time invasive and non-invasive respiratory support.

## Discussion

This is the first study to show the beneficial effects of ERT on the survival of adult patients with Pompe disease, a clinically meaningful finding especially given the slowly progressive disease course and relatively short treatment period. ERT in Pompe disease was initially approved for all patients in the United States and Europe on the basis of the prolonged survival of severely affected infants with classic Pompe disease and later by the significant gain in walking distance and stabilized pulmonary function in adult patients [8,9,20].

We observed the significant effect of ERT on survival as part of an international observational study that provided access to data from 283 adult patients with Pompe disease. Because most adult patients with Pompe disease eventually die of respiratory failure [1,7], the beneficial effect of ERT on survival is likely to be related to its positive effect on pulmonary function. The hazard ratio of 0.41 indicates that given a specific point in time a patient on ERT has a 59% smaller chance of dying than someone not on ERT. The interpretation of this effect over the entire follow-up period is, however, not intuitive. Because of the time-dependent nature of the analysis it was not possible to estimate the additional years of life gained under ERT. However, we have made ‘ad hoc’ calculations assuming the adjusted HR can be interpreted as a relative risk over approximately 4 years median and 8 years maximum follow-up (from start of treatment). Using the overall raw death rate as an estimate of the raw death rate of the treated population (16%, 46/283), eight years of ERT would result in 1 year of life gained.

Our estimate should be conservative as many patients in our cohort started treatment late in their disease course and ERT was not registered until 2006. It has been hypothesized that starting treatment early in the disease course results in a better clinical outcome [9-11,13]. Indeed, all patients (with one exception) in the ERT group who subsequently died were dependent on a wheelchair and/or a ventilator and thus had a very advanced stage of disease when they first received ERT. The effect of ERT on survival may therefore be greater if treatment is initiated earlier. In addition, the positive effect of ERT observed in this analysis overall suggests that patients with advanced disease may also benefit from treatment. Further research is required to investigate the association between disease severity and treatment effect.

Collecting sufficient data to demonstrate treatment efficacy is a challenge in rare diseases. Demonstrating improved survival is particularly difficult in a slowly progressive disease such as adult Pompe disease. The opportunity to compare the natural course of Pompe disease with the disease course following ERT highlights the importance of our unique database. In this cohort, the majority of patients switched from being untreated to being treated with ERT during follow-up. Therefore, we conducted Cox regression analyses using ERT as a time-dependent variable, which was considered the most suitable method because it prevents “immortal time” bias. Immortal time bias refers to a period of follow-up or observation time during which death cannot occur, [21] which in our study would be the time until ERT became available for the patients who survived to that time point and received treatment afterward.

Our study was observational and did not have the scientific rigor of a randomized controlled trial, which is generally considered the most appropriate method for comparing the effects of (alternative) treatments. However, a placebo-controlled randomized clinical trial over as many years as in our observational study is not possible nor is it ethically acceptable to conduct; our prospective follow-up study provided a valid alternative [22]. In addition, a clinical trial requires very strict inclusion and exclusion criteria and may not be representative of the entire adult Pompe patient population. Recruitment through a patient organization could also result in more or less severely affected patients being excluded. However, demographic and clinical characteristics of our study population show that patients were included across the entire disease spectrum and were representative of the whole patient population. A number of confounders were adjusted for in the analysis, including age, gender and disease severity, as well as country of residence to capture country specific differences such as variation in approaches to care. Selection bias was further minimized through the time dependent nature of the analysis, as the same patient could contribute to both the untreated and treated period.

Our results were robust across the different models, strengthening our conclusion that ERT positively influences patient survival. We used the equivalent of an intent-to-treat approach, because this is the standard method of analysis used to assess treatment effects in clinical trials. This cohort included 19 patients who stopped treatment during follow-up, and it may be perceived as unfair to include their time after discontinuation of ERT as “time on treatment”. An additional analysis in which patients who discontinued treatment were followed only until the end of treatment provided similar results to the intent-to-treat analyses, but with greater statistical significance.

## Conclusions

Enzyme replacement therapy with alglucosidase alfa is currently the only approved disease-specific therapy for Pompe disease. Although not curative, it has changed patients’ perspectives through demonstrated improvements in muscle strength, pulmonary function, and other clinical parameters. Our novel findings reported here show ERT to also have a positive impact on survival of adult patients with Pompe disease. This may be considered an important and clinically meaningful observation, which is particularly relevant with respect to the recent discussion concerning the reimbursement of ultra-orphan drugs.

## Competing interests

Research on Pompe disease at Erasmus MC is financially supported by the following parties: ZonMw - the Netherlands Organisation for Health Research and Development [project no. 152001005]; the Dutch TI Pharma initiative “Sustainable Orphan Drug Development through Registries and Monitoring (T6-208); “EUCLYD - a European Consortium for Lysosomal Storage Diseases” (health F2/2008 grant agreement 201678); the Prinses Beatrix Fonds [project no. OP07-08]; and Genzyme Corporation, Cambridge, MA, USA. Since August 2004, A.T. vd.P. and A.J.J.R., have provided consulting services for Genzyme Corp, Cambridge, MA, USA, under an agreement between Genzyme Corporation and Erasmus MC, Rotterdam, the Netherlands. This agreement also covers financial support for Erasmus MC to pursue research in the field of Pompe disease. Erasmus MC and inventors of the method of treatment of Pompe disease by enzyme replacement therapy receive royalty payments pursuant to Erasmus MC policy on inventions, patents and technology transfer. The authors confirm independence from the funders; the content of the article has not been influenced by the funders.

## Authors’ contributions

DG was involved in the study design, and data collection, performed the statistical analyses and drafted the manuscript. MEK, IP and RBD supervised the statistical analyses, made substantial contributions to the interpretation of the results, and critically revised the manuscript. MLCH was involved in the design of the study and data collection and critically reviewed the manuscript. PAvD, AJJR, and ATvdP conceived of the study, participated in its design and in the interpretation of results, and coordinated the drafting of the manuscript. All authors read and approved the manuscript.
